# Tracking pulmonary gas exchange by breathing control during exercise: role of muscle blood flow

**DOI:** 10.1113/jphysiol.2013.261396

**Published:** 2013-10-11

**Authors:** Philippe Haouzi

**Affiliations:** Department of Medicine, Division of Pulmonary and Critical Care Medicine, Penn State University College of MedicineHershey, PA, USA

## Abstract

Populations of group III and IV muscle afferent fibres located in the adventitia of the small vessels appear to respond to the level of venular distension and to recruitment of the vascular bed within the skeletal muscles. The CNS could thus be informed on the level of muscle hyperaemia when the metabolic rate varies. As a result, the magnitude and kinetics of the change in peripheral gas exchange – which translates into pulmonary gas exchange – can be sensed. We present the view that the respiratory control system uses these sources of information of vascular origin, among the numerous inputs produced by exercise, as a marker of the metabolic strain imposed on the circulatory and the ventilatory systems, resulting in an apparent matching between pulmonary gas exchange and alveolar ventilation.

This review presents and discusses some of the experimental evidence gathered over the last few years (Haouzi *et al*. 2009; Forster *et al*. 1958) supporting the role for a signal originating from the muscle circulation, related to the local metabolic demand, which contributes to the matching between ventilation and pulmonary gas exchange during exercise (Dejours, 1977; Whipp, 1978; Whipp & Ward, 1990; Forster *et al*. 1958).

The term ‘exercise’ refers here to any muscular activity associated with dynamic contractions, consisting of a succession of rhythmic contractions and relaxations such as walking, running or cycling and resulting in an increase in metabolic rate. Static, or isometric, contractions which are to be regarded as an ‘effort’ (Dejours, 1975), i.e. sustaining a constant load such as carrying a weight, produce ventilatory responses different, both qualitatively and quantitatively, from those observed during dynamic exercise (Poole *et al*. 1990b; Imms & Mehta, 1993). These responses will not be discussed here.

The inability of the cardiovascular system to appropriately increase an already high resting level of O_2_ supply to the tissues and CO_2_ transport to the lungs requires blood flow to be redistributed toward the exercising muscle wherein muscle metabolic rate increases (Astrand & Rodahl, 2010; Guyton, 1978; Laughlin *et al*. 1993). The theory presented in this paper is that the changes in vascular resistance within the exercising muscles produce a specific signal that may prove to be essential for the ‘respiratory neurons’ involved in the coupling between ventilation and peripheral/pulmonary gas exchange. The elements supporting the ‘vascular distension hypothesis’ are briefly described. For more details the reader can refer to the following reviews (Haouzi *et al*. 2009; Haouzi, 2007; Forster *et al*. 1958). Finally, a model will be presented on how this information may be used by the CNS to produce a matching between *alveolar* ventilation (but not *minute* ventilation) and the pulmonary gas exchange rate.

## Setting the scene: rate of O_2_ and CO_2_ transport by the blood and muscle blood flow redistribution

The transfer of molecules of oxygen from the atmosphere to the mitochondria of millions of cells to eventually ‘feed’ the electron chain, along with the elimination of the molecules of CO_2_ produced in the process, relies on a profound interaction between the circulatory and the ventilatory systems (Dejours, 1959; Astrand & Rodahl, 2010).

In resting mammals, including humans, the rate of O_2_ delivery by the arterial blood (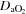 ) – the product of the cardiac output and the arterial O_2_ concentrations (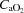 ) – is several fold the rate of body O_2_ consumption (Astrand & Rodahl, 2010; Dejours, 1981, 1988). For instance, in an average-sized adult human, resting 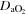 is about 1000 ml min^−1^, assuming an arterial O_2_ content of 200 ml l^−1^ and a cardiac output of 5 l min^−1^ (Guyton *et al*. 1973), while resting 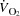 ranges between 250 and 300 ml min^−1^ (Dejours, 1964). The volume of O_2_ delivered per minute in the arterial tree is therefore 3–4 times the volume of O_2_ consumed over the same time. As a consequence, 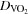 (the rate of O_2_ delivery back to the lungs) is only 25% less than 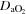 (Dejours, 1964). Incidentally, in air-breathing animals, the rate at which CO_2_ is transported by the venous system (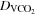 ) is also 3–4 times its rate of production: There is, in addition to the CO_2_ produced by all the tissues, about 1 litre min^−1^ of CO_2_ coming for the arterial side which must be transported by the venous system towards the lungs (Dejours, 1981). The latter only eliminates, through alveolar ventilation, the equivalent of the endogenous production of CO_2_, 200–250 ml min^−1^ at rest.

During a dynamic exercise, muscle 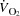 and 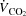 can increase up 20-fold (Astrand & Rodahl, 2010; Dejours, 1964); 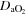 to the muscles must therefore rise to prevent or limit a reduction in muscle 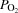. Similarly, as 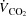 must rise by about the same amount as 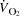 and at a similar rate – or even at a higher rate during heavy exercise – the only way to limit the rise in muscle CO_2_ is for the cardiovascular system to allow for an increase in blood flow to (and therefore from) the metabolically active tissues. As O_2_ extraction and cardiac output can, at the very best, increase by 3 times each at the peak of a maximal exercise in a trained athlete (Astrand & Rodahl, 2010; Dejours, 1964), a redistribution of blood flow towards the exercising muscles (Saltin *et al*. 1974; Rowell, 1981; Rowell & O'Leary, 1988; Laughlin, 1993) is the only mechanism through which O_2_ delivery rate can match the increased rate of O_2_ demand in the muscles. This redistribution of muscle blood flow involves a decrease in vascular resistance in the metabolically active territories, while peripheral conductance decreases in the non-exercising muscles and tissues, for example the skin or the gastrointestinal system (Mitchell, 1983).

## The challenge of exercise-induced hyperpnoea: ‘matching’ peripheral/pulmonary gas exchange

The involvement of the ventilatory system during exercise should be viewed as one of the components of the adjustments described in the previous paragraph, as: (1) the level of breathing must cope with the consequences of an increase in pulmonary gas exchange, (Whipp, 1978; Forster *et al*. 1958), a direct effect of the increase in muscle gas exchange; and (2) the magnitude of the ventilatory response dictates the level of alveolar – and thus arterial – O_2_ and CO_2_ partial pressures, and contributes to maintaining an adequate rate of O_2_ delivery and CO_2_ removal (Whipp & Ward, 1992; Haouzi, 2007). Keeping 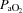 and 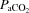 constant will certainly limit the ‘circulatory cost’ required to raise 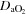 to the exercising muscles.

As the circulatory system is a closed circuit (if, as a first approach, one ignores venous capacitance), the changes in pulmonary blood flow and pulmonary gas exchange mirror the averaged gas exchange occurring in all of the peripheral tissues – unless disrupted by experimental means (see below for the effects of venous *vs*. arterial occlusion). Consequently, the convection of a gas in the regions of the lungs wherein gas exchange takes place, i.e. the alveolar regions, must increase to prevent alveolar 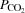 from rising and alveolar 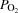 from dropping in proportion to the rate at which peripheral gas exchange increases. To get a quantitative idea of the importance of the adjustment of the convection of gas to the pulmonary gas exchange, consider that a walking human increases O_2_ consumption (and CO_2_ production) and thus lung gas exchange by about 3 times. As 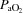 = 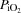 – *k*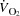 /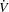
_*A*_ and 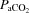 = 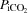 + *k*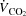 /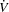
_A_, 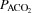 (and 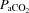 ) will increase by 3 times (up to 120 Torr!) and 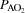 (and 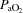 ) would be close to zero, if 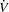
_A_ did not increase during this very moderate form of exercise.

**Figure 1:**
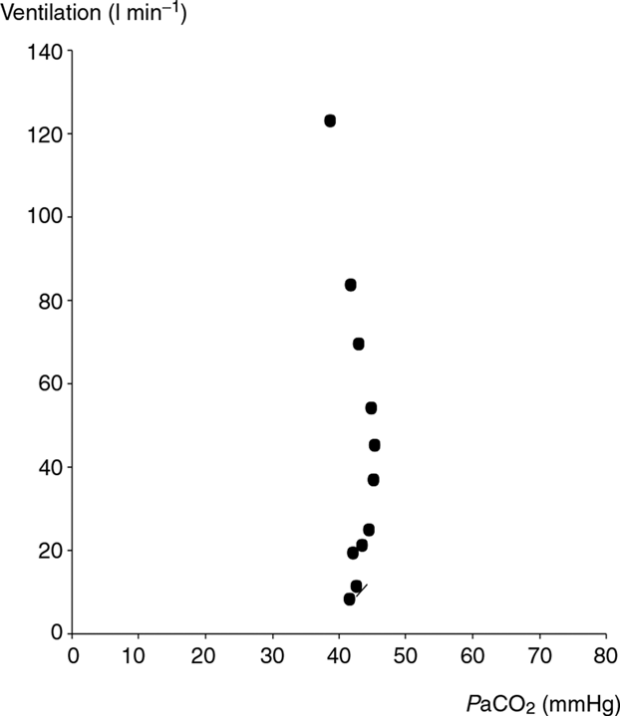
Note that ventilation increases with no increase in 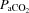 during a moderate level of exercise, while hypocapnia develops at a higher level of work rate and breathing.

**Figure 2:**
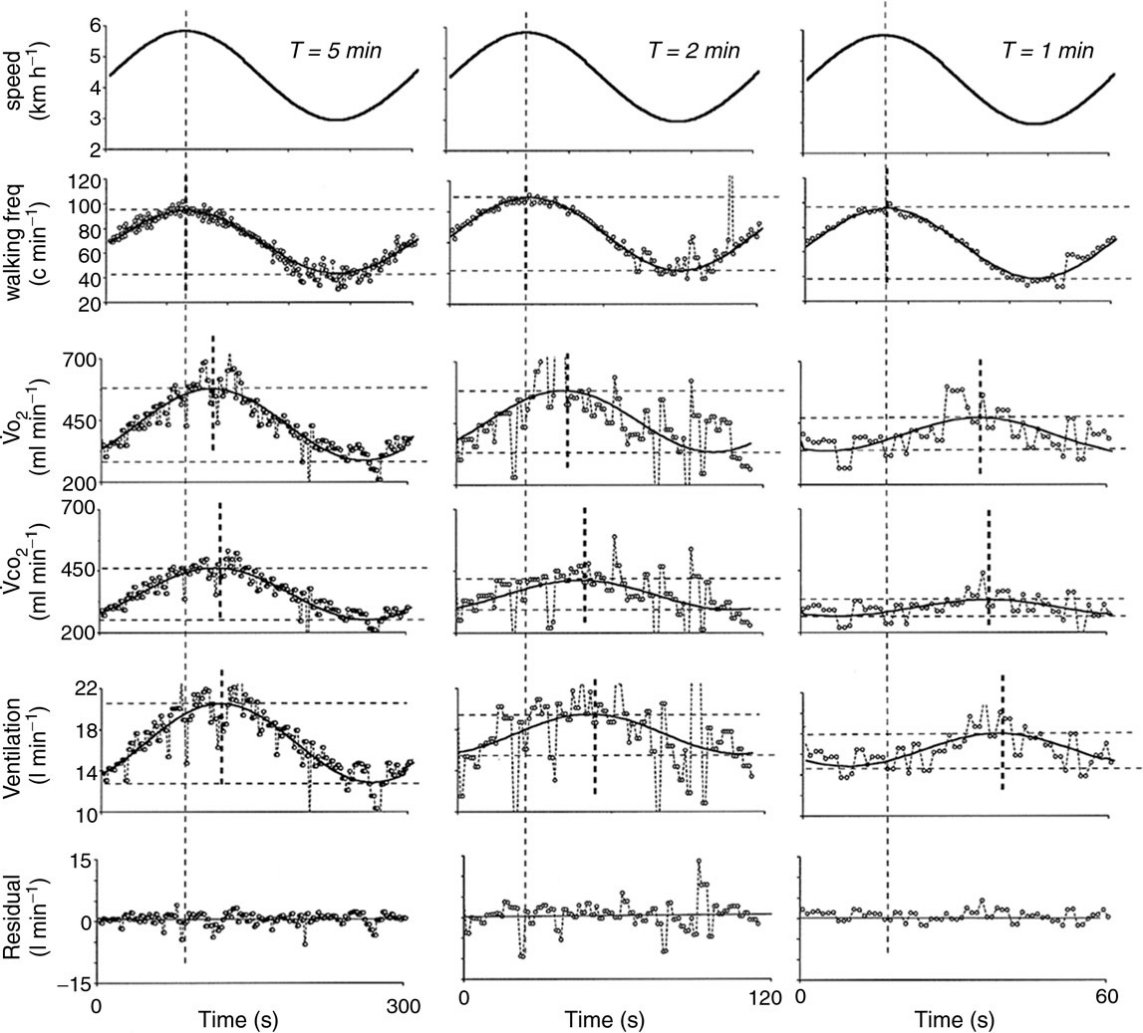
As the period of change in walking speed decreases, the pulmonary gas exchange response becomes dissociated from the motor activity due to a slower time constant response than the locomotor activity, which follows very precisely the change in walking speed. Minute ventilation follows the change in pulmonary gas exchange, with a reduction in amplitude and an increase in phase lag despite unchanged locomotor and motor control. Similar results have long been reported in humans during cycling exercise (Casaburi *et al*. 1977).

As shown in Figs 1 and 2, not only does minute ventilation increase in proportion to the pulmonary gas exchange, but this ventilatory adjustment has kinetics which seem to ‘follow’ that of 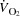 or 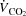 during both the onset of and the recovery from any form of dynamic exercise. This remains true whether a step, an impulse, a ramp or a fluctuating change in work load is applied (Fujihara *et al*. 2012, 1991; Casaburi *et al*. 1977; Whipp, 1982, 1978; Haouzi *et al*. 2011). An important point needs to be clarified about 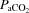 homeostatis during exercise: the ventilation tracks in all types of exercise factors related or proportional to the pulmonary gas exchange, but: (1) it is not the pulmonary gas exchange by itself which seems to contain the signal driving breathing; and (2) because the ventilatory kinetics are slower than that of gas exchange, a transient, albeit small, change in 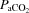 does occur in the unsteady state conditions of a step or sinusoidal change in work load (Whipp, 1982). The fundamental question of exercise-induced hyperpnoea is therefore to address the mechanisms accounting for the ventilatory–gas exchange matching. In steady-state and unsteady-state conditions, the result of this matching is to prevent (or limit) the rise in 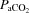 (and decrease in 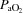 ), which would result from an increase in 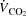 (or 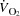 ).

Finally, the evolution of the lung anatomy in air-breathing vertebrates has led to the development of a significant pulmonary dead space (Dejours, 1981), wherein gas circulates between the alveolar regions and the atmosphere without being exchanged. It is alveolar, and not minute, ventilation that controls the levels of CO_2_ and O_2_ to be exchanged with the blood. The puzzling question is how could alveolar ventilation be regulated while it is minute ventilation (a tidal volume at a given frequency) that is generated by the respiratory neurons. This certainly adds some complexity to the conundrum represented by the mechanism of exercise-induced hyperpnoea.

## The ‘peripheral vascular distension hypothesis’

The challenge is not to propose a theory that can account for any rise in ventilation (indeed multiple structures evoked during an exercise can stimulate breathing, for example muscle receptors, chemoreception and supra-medullary structures), but rather to propose a mechanism that can account for the magnitude and time course of the ventilatory response and its link to the pulmonary gas exchange (Whipp & Ward, 1991).

Over the last few years, our group, following the original work of Huszczuk *et al*. (1976), has defended the view that one of the pathways through which the control of breathing may follow metabolic changes in the lungs is to follow metabolic changes in the muscle but based on the neural monitoring of the peripheral vascular events (Haouzi *et al*. 2009).

Briefly, skeletal muscle afferent fibres are traditionally divided into four groups according to their conduction velocity. The group III and IV muscle afferent fibres (small myelinated or unmyelinated fibres) do increase breathing (McCloskey & Mitchell, 1978; Amann *et al*. 2010). The natural stimuli of these endings, besides ‘painful’ stimuli, include the mechanical distortion of their receptive field (mechanoreception), the accumulation of ‘metabolic by-products’ of the contractions and an increase in local temperature (Hertel *et al*. 1993; Kaufman *et al*. 1982, 1989; Mense, 1972).

**Figure 3:**
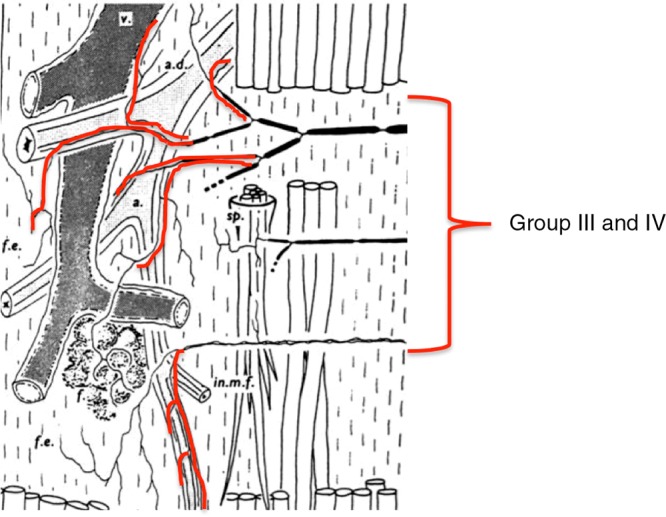
Many group III and IV afferents fibers can be found in association with arterioles and venous structures.

**Figure 4:**
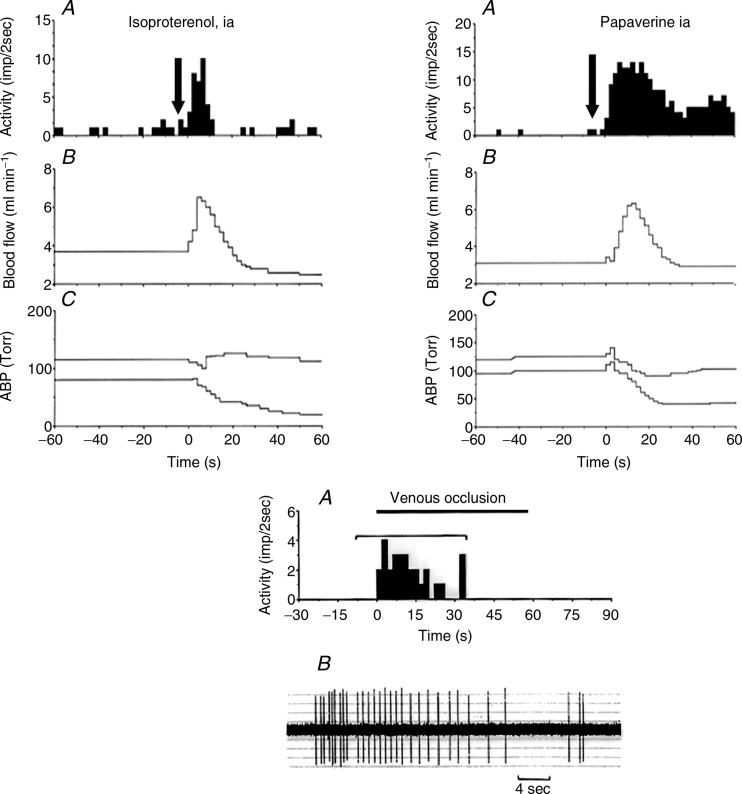
Upper panels: *A*, histogram of activity. *B*, popliteal blood flow. *C*, arterial blood pressure (ABP). Arrows indicate the time of injection. Note that the fibre shown at the left responds immediately as soon as blood flow increases. No change was observed during vehicle injection. Lower panel: response to venous occlusion of a group IV afferent fibre, which also responded to papaverine and venous contraction (not shown). The response to the occlusion of the vena cava suggests that the receptive field of this ending is located on the venular side of the muscle circulation.

Stacey (1990) had already noted that although there is a large range of termination sites within the muscle structures, many group III and IV fibres are originating from the blood vessel adventitia, including the arterioles and venules (Fig. 3). Also, Von Düring & Andres (2001) found striking anatomical relationships between muscle group IV endings and the vessels in the cat. In keeping with these anatomical findings, we found that a population of these fibres did respond to the distension of the vessels, predominantly at venular level, and could monitor muscle blood flow by encoding the degree of recruitment of the post-capillary network (Haouzi *et al*. 2004b). For instance, in 60 slowly conducting afferent fibres present in the dorsal roots coming from the cat triceps surae, we found that 31% of group IV and 15% of group III nerves were stimulated by the vascular smooth muscle relaxant papaverine (2–2.5 mg kg^−1^; Fig. 4). Sixty-two per cent of them were also stimulated by isoproterenol, and more than half of the fibres that were stimulated by papaverine were also stimulated during an occlusion of the vena cava, suggesting that these fibres are located within or close to the venous or venular structures. Finally, a large number of group IV fibres respond to both dynamic contractions and venous distension or vasodilation (Fig. 4).

**Figure 5:**
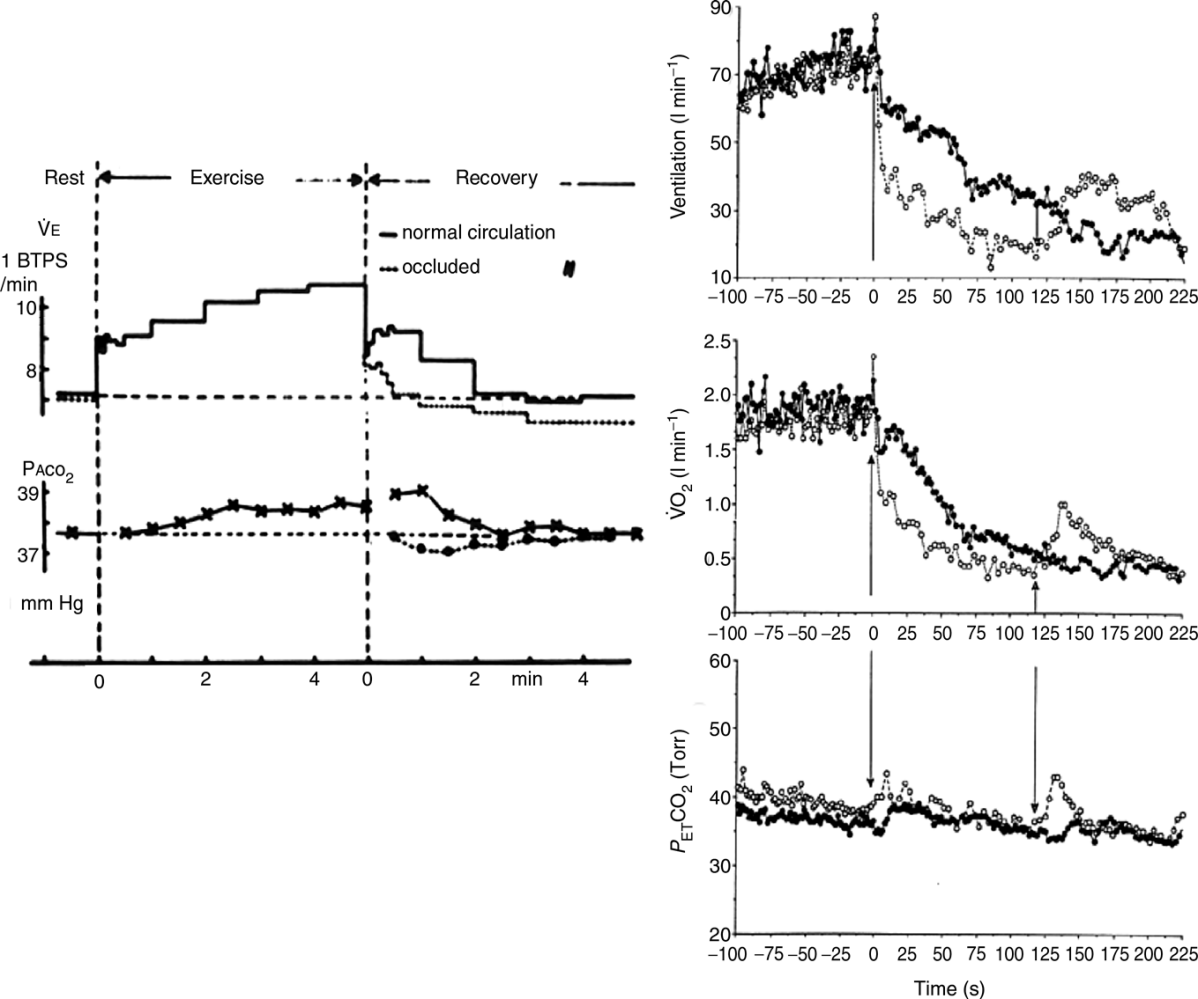
Left panel: example of the breath-by-breath minute ventilation (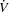
_E_), and alveolar 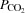 during recovery from a light level of exercise with intact circulation and while impeding the circulation to the post-exercising legs (Dejours *et al*. 1990). Right panel: Minute ventilation (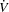
_E_), O_2_ uptake (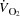 ), and end-tidal 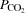 (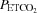 ) during recovery (filled symbols) from a constant work rate cyclo-ergometer exercise performed above the lactate threshold and during recovery with cuffs inflated for 2 min around the upper thigh (open symbols) (adapted from Haouzi *et al*. 1997). The first arrow indicates the cessation of exercise and cuff inflation; the second arrow indicates the moment of occlusion release. Note that in both studies, the normal ventilatory decline was depressed during cuff occlusion, resulting a large ventilatory deficit, despite expected accumulation of metabolites in the muscle circulation.

In accordance with these neurophysiological findings, reflexes triggering ventilation changes could be elicited when altering muscle circulation. For instance, injection of a vasodilatory agent into the isolated hindlimb circulation of a sheep stimulates breathing (Haouzi *et al*. 1999). This occurs well before the drug could have reached the central circulation and, thus, could have affected the arterial chemo- or baroreceptors. Conversely, total obstruction of the blood flow to and from the limbs at rest or at the cessation of dynamic exercise (Fig. 5) does not stimulate, but actually decreases, ventilation much faster than in control conditions, which decline towards resting levels in humans and in animals (Dejours *et al*. 1990*b*; Haouzi *et al*. 1997; Fukuba *et al*. 1973a). However, impeding, by intravascular occlusion, the circulation from (venous side) or to (arterial side) the hindlimbs during electrically induced muscle contractions in dogs (Huszczuk *et al*. 1976) leads to opposite ventilatory outcomes, regardless of the circulatory changes occurring in the central circulation. Indeed, despite a similar reduction in O_2_ uptake and blood pressure resulting from obstruction of the caudal vena cava or the distal abdominal aorta, ventilation typically rises when the venous side is occluded and decreases when the arterial side is impeded during exercise (Huszczuk *et al*. 1976; Haouzi *et al*. 2009). Every condition associated with an impediment of the arterial supply prevents the normal 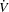
_E_ response produced both at the onset and during the steady-state response to dynamic contractions in various animal models. A similar reduction in 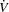
_E_ response can be observed in patients with peripheral vascular disease of the lower extremities when walking on a treadmill, below their pain threshold (Haouzi *et al*. 2007). Such reduction in breathing during exercise is observed despite all of the other stimuli being present (e.g. control of movements and intensity of contractions). These results also fit with a puzzling observation that whenever the motor act is dissociated from metabolic or gas exchange changes, the strategy adopted by the ventilatory control system is not to follow factors related to the motor activity but to follow, in a systematic and predictive way (Haouzi *et al*. 2011), factors proportional or related to some of the changes associated with the rate at which CO_2_ is exchanged in the lungs (Fig. 2). Any concept that neglects this crucial observation cannot account for the fundamental mechanism of 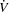
_E_ control in exercise, as this approach preserves all other inputs to the CNS (Whipp & Ward, 1990).

**Figure 6:**
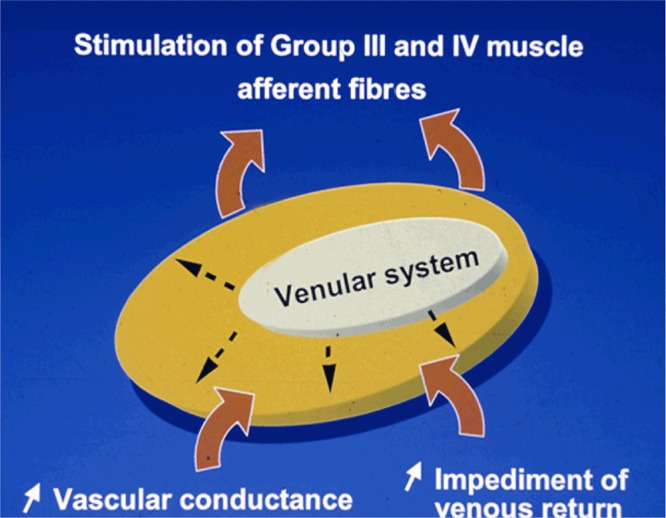
Any event distending the venular system (venous occlusion, hyperemia or mechanical deformation of the receptive field in keeping with volume of blood present) can in turn stimulate group III and IV endings.

## How to understand the apparent matching between alveolar ventilation and pulmonary gas exchange in exercise

The question is how much the system described in the above section can contribute to the ventilatory response to exercise, wherein minute ventilation can increase by more than 100 l min^−1^ (Dejours, 1959)? Answering this question requires an understanding of how the structures in the CNS involved in breathing control process the multitude of available sources of information reaching the medullary and supra-medullary structures. Although the neurophysiological basis to understand this neuronal processing is still lacking, precious information can still be obtained from what we already know on the 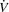
_E_ response to exercise, as presented in the following section.

Exercise is a complex physiological condition, which combines the consequences of a motor activity with those related to an increase in metabolism. Motor activity involves the control and effects of elementary muscle contractions and movements (gate, cycling); the structures implicated in this process include specific cortical (Thornton *et al*. 2001; 1969; Williamson *et al*. 1981) or subcorticical regions (Eldridge & Waldrop, 1994; Eldridge, 1957) as well as peripheral inputs related to contractions or movement (Kniffki *et al*. 1987; Mense, 1999; Kaufman *et al*. 1989). The metabolic changes can trigger many different inputs: (1) an increase in CO_2_ venous content and venous blood flow (Greco *et al*. 1973b; Bennett *et al*. 2010) and a decrease in the mixed venous O_2_ content which could in turn affect the arterial chemoreceptors (Phillipson *et al*. 1990a); (2) a change in the chemical composition in the muscles (Mense & Stahnke, 1996; Kaufman & Rybicki, 2002) affecting muscle afferents; (3) an increase in local and systemic temperature (Dejours *et al*. 1963; Budzinska, 1984; Hertel *et al*. 1993); and (4) an increase in systemic and muscle blood flow potentially stimulating receptors located in the central (Jones *et al*. 1983; Huszczuk *et al*. 2012) as well as peripheral circulation (Haouzi, 2006), while blood flow decreases in many non-exercising tissues. Despite the fact that all of these inputs, with their different magnitude and time constants, can ‘reach’ the CNS in an almost infinite number of combinations and can all increase breathing separately, the ventilatory response to exercise seems to follow the metabolism in a rather simple and perfectly predictive manner (Whipp, 1982; Whipp *et al*. 1998). The strategy used by the ‘respiratory neurons’ seems to rely on properties that have more to do with the selection of information and pattern recognition than to the integration of individual inputs. In other words, all of the signals produced by exercise are not *qualitatively* important in translating into an increase in breathing. According to this view, the CNS does not respond in proportion to (or as a function of) a given stimulus, which could be predicted by, for instance, the elementary 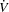
_E_ response to CO_2_, hypoxia, a muscle contraction or a change in blood flow. The respiratory control system seems to use all of the various sources of information to determine that an exercise is actually being performed and to select the most relevant sources of information for adjusting breathing (see Fig. 2). Tracking the change in peripheral gas exchange, via the change in circulation, may well be part of this strategy. This is, after all, what the response to ‘sinusoidal’ or ‘impulse’ exercise reveals (Whipp, 1978; Haouzi, 2007). Similarly, when there are antagonist sources of information, such as dissociating venous return to the lungs from the arterial supply, the strategy adopted by the CNS always seems to follow factors associated with an increase in the local vascular response, as if it were the only relevant information for controlling breathing related to exercise (for discussion see Haouzi, 2007). By contrast, the respiratory control system may not be able to recognize that an exercise is being performed when different conditions are created, such as in patients with peripheral vascular disease (Haouzi *et al*. 2007) or, even more dramatically, during a cardiac arrest, resulting in a completely novel and unpredictable 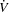
_E_ response (Haouzi *et al*. 1996). With such a view, the matching between 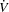
_A_ and pulmonary gas exchange cannot be predicted by any signal considered individually (Haouzi *et al*. 1996)

Finally, the debate over the fact that it is 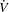
_A_ and not 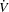
_E_ that appears to be regulated while it is 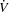
_E_ which is generated could be understood by the constraint imposed on the respiratory system by the ‘non-proportional’ relationship between the amplitude and the duration of any breath (Haouzi *et al*. 2004a; Haouzi & Bell, 2009; Haouzi, 1987). Indeed, it is the change in dead space ventilation (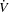
_D_) and not in dead space (*V*_D_) that can disrupt blood gas during exercise; we have recently proposed that there is an obligatory relationship between the amplitude and duration of any breath – this relationship is not proportional but has a positive intercept of the magnitude of *V*_D_, which results in an apparent regulation of 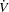
_A_ (Haouzi *et al*. 2004a; Haouzi & Bell, 2009) whatever the level of breathing frequency is adopted. Following such relationship, while it is minute ventilation (the product of a tidal volume by the breathing frequency) that is ‘generated’ by the medullary and spinal respiratory moto-neurons (Mitchell, 1992), it is
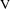
_A_ that is being regulated.

## Concluding remarks

The view presented in this paper is that the volume of blood at the venular level in the muscles could constitute a crucial stimulus informing the CNS that metabolism is changing in peripheral tissue. The CNS produces a ventilatory output that follows the levels of gas exchange but which, intriguingly, appears to neglect other signals, at least in terms of their kinetics. The matching between alveolar ventilation and pulmonary gas exchange observed during exercise may result from a complex process in which the ventilatory strategy chosen by the CNS is determined by (1) the magnitude and kinetics of the vascular response in the muscles; (2) all other inputs produced by exercise, related or not to gas exchange or to one of its circulatory surrogates, which inform the respiratory neurons that an exercise is being performed (see Fig. 2); and (3) the fundamental relationship between the amplitude and the duration of any breath, which results in a regulation of alveolar rather than minute ventilation.

## References

[b1] Amann M, Blain GM, Proctor LT, Sebranek JJ, Pegelow DF, Dempsey JA Group III and IV muscle afferents contribute to ventilatory and cardiovascular response to rhythmic exercise in humans. J Appl Physiol.

[b2] Astrand, Rodahl Textbook of Work Physiology.

[b3] Bennett FM, Tallman RD, Grodins FS (2010). Role of VCO2 in control of breathing of awake exercising dogs. J Appl Physiol.

[b4] Budzinska K (1984). Effects of hyperthermia and stimulation of the hypothalamus on the activity of the phrenic nerve in hypo- normo- and hypercapnic rabbits. Acta Neurobiol Exp (Wars).

[b5] Casaburi R, Whipp BJ, Wasserman K, Beaver WL, Koyal SN (1977). Ventilatory and gas exchange dynamics in response to sinusoidal work. J Appl Physiol.

[b6] Dejours P (1975). [Regulation of ventilation during muscular exercise in man.]. J. Physiol (Paris).

[b7] Dejours P, Cunningham DJC, Lloyd BB (1977). The regulation of breathing during muscular exercise in man: A neuro-humoral theory. The Regulation of Human Respiration.

[b8] Dejours P, Fenn WO, Rahn H (1959). Control of respiration in muscular exercise. Handbook of Physiology, Section 3, Volume I, Chapter 25.

[b9] Dejours P (1981). Principles of Comparative Respiratory Physiology.

[b10] Dejours P (1988). Respiration in Water and Air: Adaptations-Regulations-Evolution.

[b11] Dejours P (1964). Comparative aspects of maximal oxygen consumption. Respir Physiol.

[b12] Dejours P, Mithoefer JC, Raynaud J (1990). Evidence against the existence of specific ventilatory chemoreceptors in the legs. J Appl Physiol.

[b13] Dejours P, Teillac A, Girard F, Lacaisse A (1963). [Study of the role of moderate central hyperthermia in the regulation of ventilation during muscular exercise in man.]. Rev Fr Etud Clin Biol.

[b14] Eldridge FL (1957). Central integration of mechanisms in exercise hyperpnea. Med Sci Sports Exerc.

[b15] Eldridge FL, Waldrop TG (1994). Neural Control of Breathing During Exercise.

[b16] Forster HV, Haouzi P, Dempsey JA (1958). Control of breathing during exercise. Compr Physiol.

[b17] Fujihara Y, Hildebrandt J, Hildebrandt JR (2012). Cardiorespiratory transients in exercising man. II. Linear models. J Appl Physiol.

[b18] Fujihara Y, Hildebrandt JR, Hildebrandt J (1991). Cardiorespiratory transients in exercising man. I. Tests of superposition. J Appl Physiol.

[b19] Fukuba Y, Kitano A, Hayashi N, Yoshida T, Ueoka H, Endo MY, Miura A (1973a). Effects of femoral vascular occlusion on ventilatory responses during recovery from exercise in human. Respir Physiol Neurobiol.

[b20] Greco EC, Fordyce WE, Gonzalez F, Reischl P, Grodins FS (1973b). Respiratory responses to intravenous and intrapulmonary CO_2_ in awake dogs. J Appl Physiol.

[b21] Guyton AC (1978). Human Physiology and Mechanisms of Disease.

[b22] Guyton AC, Jones CE, Coleman TG (1973). Cardiac Output and its Regulation.

[b23] Haouzi P (2007). Theories on the nature of the coupling between ventilation and gas exchange during exercise. Respir Physiol Neurobiol.

[b24] Haouzi P (2006). Venous pressure and dyspnea on exertion in cardiac failure: was Tinsley Randolph Harrison right. Respir Physiol Neurobiol.

[b25] Haouzi P (1987). Initiating inspiration outside the medulla does produce eupneic breathing. J Appl Physiol.

[b26] Haouzi P, Bell HJ (2009). Control of breathing and volitional respiratory rhythm in humans. J Appl Physiol.

[b27] Haouzi P, Chenuel B, Chalon B (2011). The control of ventilation is dissociated from locomotion during walking in sheep. J Physiol.

[b28] Haouzi P, Chenuel B, Huszczuk A (2009). Sensing vascular distension in skeletal muscle by slow conducting afferent fibers: neurophysiological basis and implication for respiratory control. J Appl Physiol.

[b29] Haouzi P, Chenuel B, Whipp BJ (2004a). Control of breathing during cortical substitution of the spontaneous automatic respiratory rhythm. Respir Physiol Neurobiol.

[b30] Haouzi P, Hill JM, Lewis BK, Kaufman MP (2004b). Responses of group III and IV muscle afferents to distension of the peripheral vascular bed. J Appl Physiol.

[b31] Haouzi P, Hirsch JJ, Marchal F, Huszczuk A (2007). Ventilatory and gas exchange response during walking in severe peripheral vascular disease. Respir Physiol.

[b32] Haouzi P, Hirsh JJ, Gille JP, Marchal F, Crance JP, Huszczuk A (1999). Papaverine injection into the hindlimb circulation stimulates ventilation in sheep. Respir Physiol.

[b33] Haouzi P, Huszczuk A, Porszasz J, Chalon B, Wasserman K, Whipp BJ (1997). Femoral vascular occlusion and ventilation during recovery from heavy exercise. Respir Physiol.

[b34] Haouzi P, Van De Louw A, Haouzi A (1996). Breathing during cardiac arrest following exercise: a new function of the respiratory system. Respir Physiol Neurobiol.

[b35] Hertel HC, Howaldt B, Mense S (1993). Responses of group IV and group III muscle afferents to thermal stimuli. Brain Res.

[b36] Huszczuk A, Jones P, Oren A, Shors E, Nery L, Whipp B, Wasserman K, Whipp B, Wiberg D (2012). Venous return and ventilatory control. Modelling and Control of Breathing.

[b37] Huszczuk A, Yeh E, Innes JA, Solarte I, Wasserman K, Whipp BJ (1976). Role of muscle perfusion and baroreception in the hyperpnea following muscle contraction in dog. Respir Physiol.

[b38] Imms FJ, Mehta D (1993). Respiratory responses to sustained isometric muscle contractions in man: the effect of muscle mass. J Physiol.

[b39] Jones PW, Huszczuk A, Wasserman K (1983). Cardiac output as a controller of ventilation through changes in right ventricular load. J Appl Physiol.

[b40] Kaufman MP, Hayes SG, Adreani CM, Pickar JG (1989). Discharge properties of group III and IV muscle afferents. Adv Exp Med Biol.

[b41] Kaufman MP, Hill JM, Pickar JG, Rotto DM (1982). Responses of group III and IV muscle afferents to mechanical and metabolic stimuli likely to occur during exercise. Respiratory Control: Central and Peripheral Mechanisms.

[b42] Kaufman MP, Rybicki KJ (2002). Discharge properties of group III and IV muscle afferents: their responses to mechanical and metabolic stimuli. Circ Res.

[b43] Kniffki K-D, Mense S, Schmidt RF (1987). Responses of group IV afferent units from skeletal muscle to stretch, contraction and chemical stimulation. Exp Brain Res.

[b44] Laughlin MH (1993). Cardiovascular response to exercise. Am J Physiol.

[b46] McCloskey DI, Mitchell JH (1978). Reflex cardiovascular and respiratory responses originating in exercising muscle. J Physiol.

[b47] Mense S, Ottoson D (1999). Slowly conducting afferent fibers from deep tissues: neurobiological properties and central nervous actions. Progress in Sensory Physiology.

[b48] Mense S (1972). Group III and IV receptors in skeletal muscle: are they specific or polymodal. Prog Brain Res.

[b49] Mense S, Stahnke M (1996). Responses in muscle afferent fibres of slow conduction velocity to contractions and ischaemia in the cat. J Physiol.

[b50] Mitchell GS (1992). Ventilatory control during exercise with increased respiratory dead space in goats. J Appl Physiol.

[b51] Mitchell JH (1983). Neural control of the circulation during exercise. Med Sci Sports Exerc.

[b52] Phillipson EA, Duffin J, Cooper JD (1990a). Critical dependence of respiratory rhythmicity on metabolic CO_2_ load. J Appl Physiol.

[b53] Poole DC, Ward SA, Whipp BJ (1990b). Control of blood-gas and acid-base status during isometric exercise in humans. J Physiol.

[b54] Rowell LB (1981). Human cardiovascular adjustments to exercise and thermal stress. Physiol Rev.

[b55] Rowell LB, O'Leary DS (1988). Reflex control of the circulation during exercise: chemoreflexes and mechanoreflexes. J Appl Physiol.

[b56] Saltin B, Blomqvist G, Mitchell JH, Johnson RL, Wildenthal K, Chapman CB (1974). Response to exercise after bed rest and after training. Circulation.

[b57] Stacey MJ (1990). Free nerve endings in skeletal muscle of the cat. J Anat.

[b58] Sun XG, Hansen JE, Stringer WW, Ting H, Wasserman K (1968). Carbon dioxide pressure–concentration relationship in arterial and mixed venous blood during exercise. J Appl Physiol.

[b59] Thornton JM, Guz A, Murphy K, Griffith AR, Pedersen DL, Kardos A, Leff A, Adams L, Casadei B, Paterson DJ (1969). Identification of higher brain centres that may encode the cardiorespiratory response to exercise in humans. J Physiol.

[b60] Thornton JM, Pederson DL, Kardos A, Guz A, Casadei B, Paterson DJ (2001). Ventilatory response to imagination of exercise and altered perception of exercise load under hypnosis. Adv Exp Med Biol.

[b61] Von Düring M, Andres K, Zenker W, Neuhuber W (2001). Topography and ultrastructure of group III and IV nerve terminals of cats gastrocnemius-soleus muscle. The Primary Afferent Neuron: A Survey of Recent Morpho-Functional Aspects.

[b62] Whipp B, Ward S, Lamarra N, Davis J, Wasserman K (1998). Parameters of ventilatory and gas exchange dynamics during exercise. J Appl Physiol.

[b63] Whipp BJ (1982). Tenets of the exercise hyperpnea and their degree of corroboration. Chest.

[b64] Whipp BJ (1978). The Control of Exercise Hyperpnea.

[b65] Whipp BJ, Ward SA, Whipp BJ, Wasserman K (1990). Coupling of ventilation to pulmonary gas exchange during exercise. Exercise: Pulmonary Physiology and Pathophysiology.

[b66] Whipp BJ, Ward SA (1991). Physiologic changes following bilateral carotid-body resection in patients with chronic obstructive pulmonary disease. Chest.

[b67] Whipp BJ, Ward SA (1992). Determinants and control of breathing during muscular exercise. Br J Sports Med.

[b68] Williamson JW, McColl R, Mathews D (1981). Evidence for central command activation of the human insular cortex during exercise. J Appl Physiol.

